# Supplementary material for: Genome-Wide Analyses of CCHC Family Genes and Their Expression Profiles under Drought Stress in Rose (Rosa chinensis)
Source: Int J Mol Sci. 2024 Aug 18;25(16):8983. doi: 10.3390/ijms25168983 (PMC11354476; doi:10.3390/ijms25168983)
Supplement: Supplementary file 1 [file ijms-25-08983-s001.zip › ijms-3159758-supplementary.pdf]

Genome-Wide Analyses of *CCHC* Family Genes and Their Expression Profiles under Drought Stress in Rose (*Rosa chinensis*)

Shijie Li, Jun Xu, Yong Cao, Jie Wu and Deqiang Zhang*

The following Supporting Information is available for this article:

Supporting Figures:

Figure S1 Sequence alignment of *RcCCHC25* and motif composition of rose CCHC proteins.

Figure S2 Collinearity analysis of the rose *CCHC* gene family with other 10 species.

Figure S3 Phylogenetic tree of *CCHC* genes across eleven species.

Figure S4 Phylogenetic tree of 450 *CCHC* genes.

Supporting Tables:

Table S1 Genome information for other 10 species.

Table S2 41 *CCHC* genes structure.

Table S3 The number of *CCHC* genes in different species.

Table S4 The Ka/Ks ratios and duplication gene pairs among rose and other species.

Table S5 Information for cis-acting elements of *RcCCHC* genes.

Table S6 Reference genome source.

Table S7 Identified duplication gene pairs in the *CCHC* gene family of Rosa chinensis.

Table S8 List of primers used in this study.

Table S9 List of proteins and gene sequences in this study.

1 Supporting Figures:

A

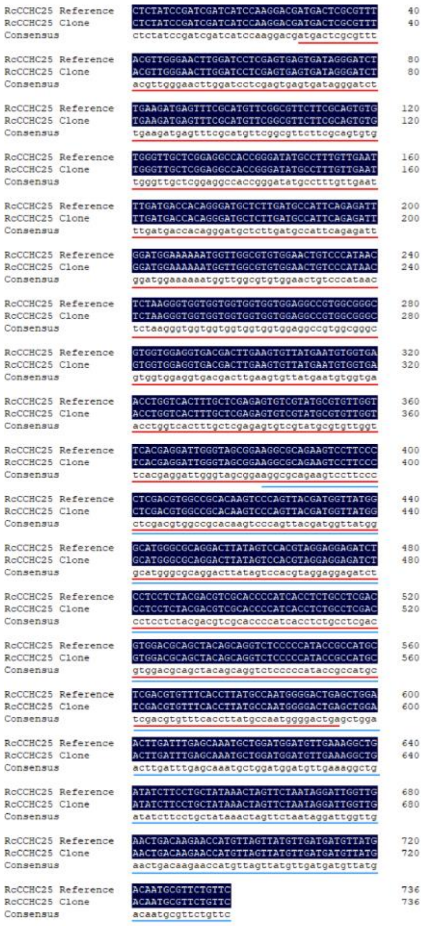

B

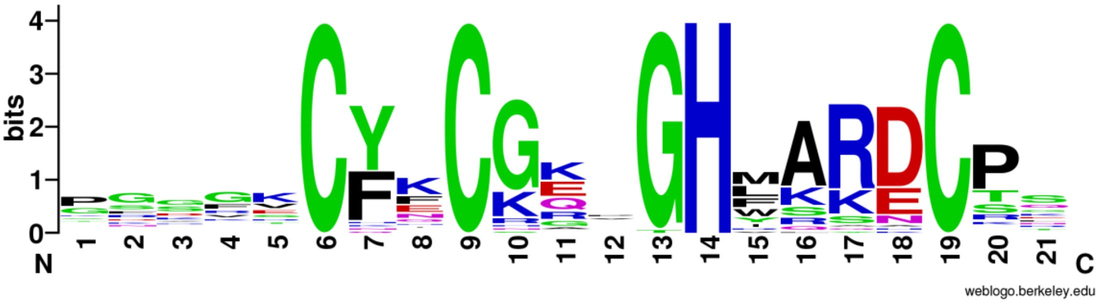

Figure S1 Sequence alignment of *RcCCHC25* and motif composition of rose CCHC proteins. A. Sequence alignment of *RcCCHC25*. Red and blue line indicate the locations of CDS region and VIGS region, respectively. **B.** Motif composition of rose CCHC proteins. The height of the letter represents the relative frequency of the corresponding amino acid appearing at that position.

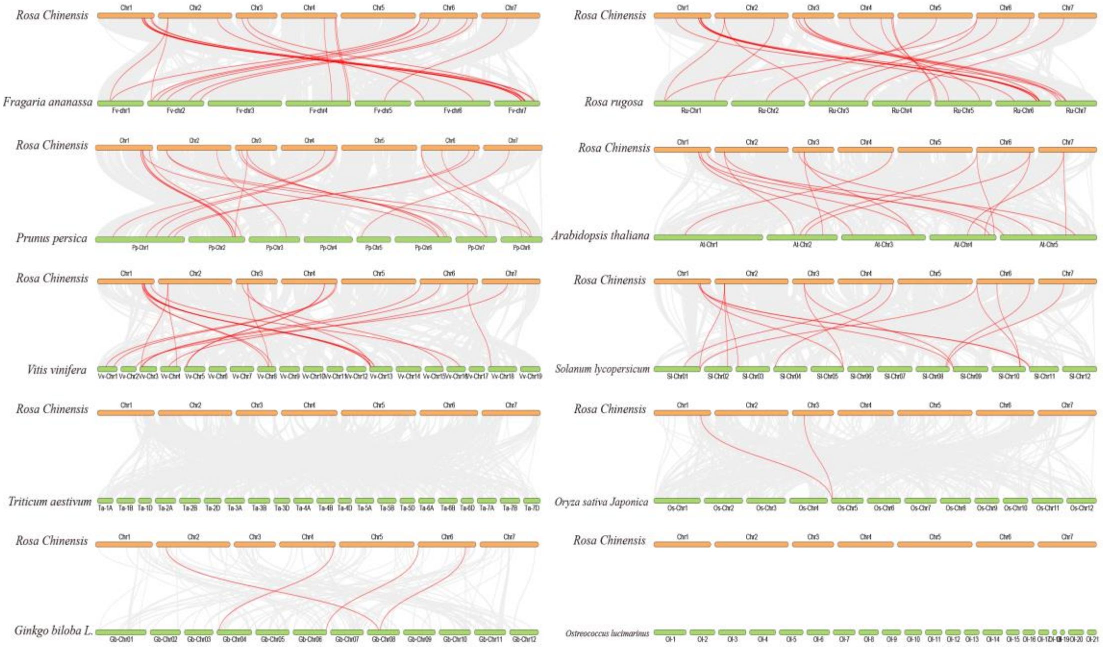

Figure S2 Collinearity analysis of the rose *CCHC* gene family with other 10 species. The grey lines and red lines represent all collinear gene pairs and collinear *CCHC* gene pairs between two species, respectively.

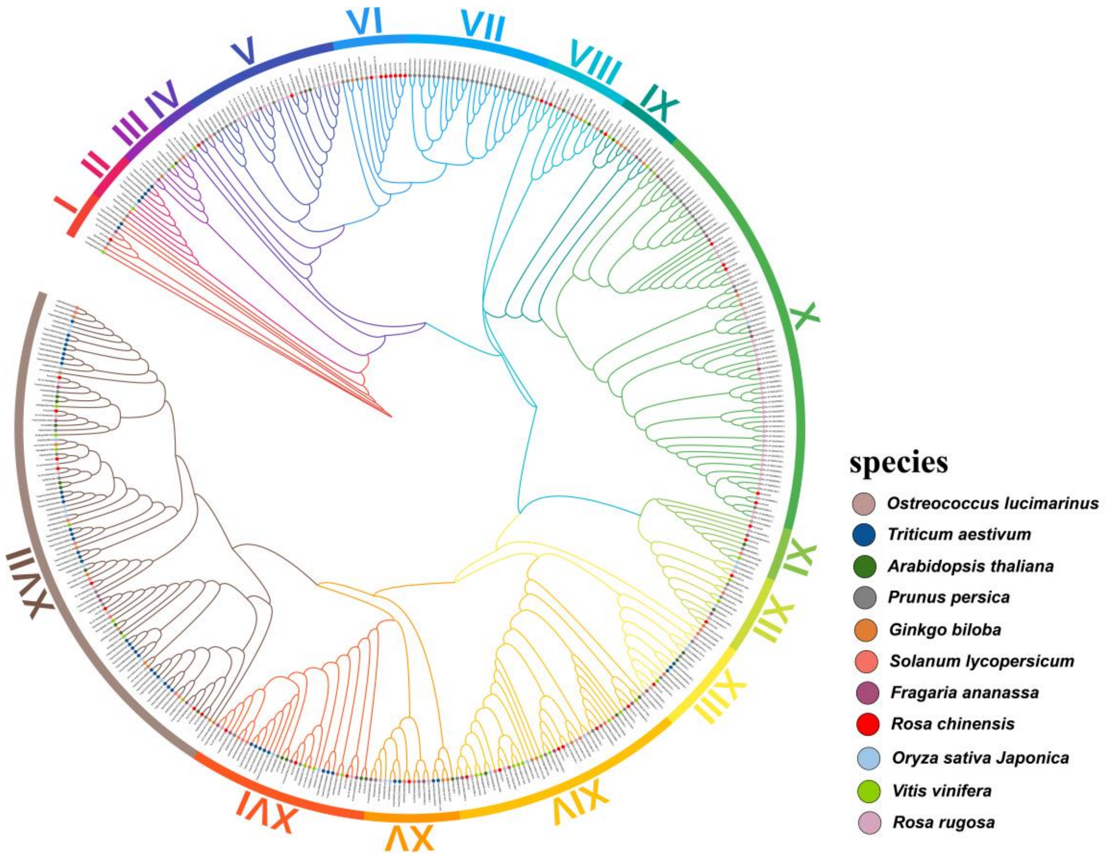

Figure S3. Phylogenetic tree of *CCHC* genes across eleven species. Each branch of the tree and the color of the outer ring denote distinct subfamilies. The names of these subfamilies are annotated along the outermost circle. Preceding each gene name, colored circles identify the species, with corresponding scientific names delineated on the right side of the figure for clarity.

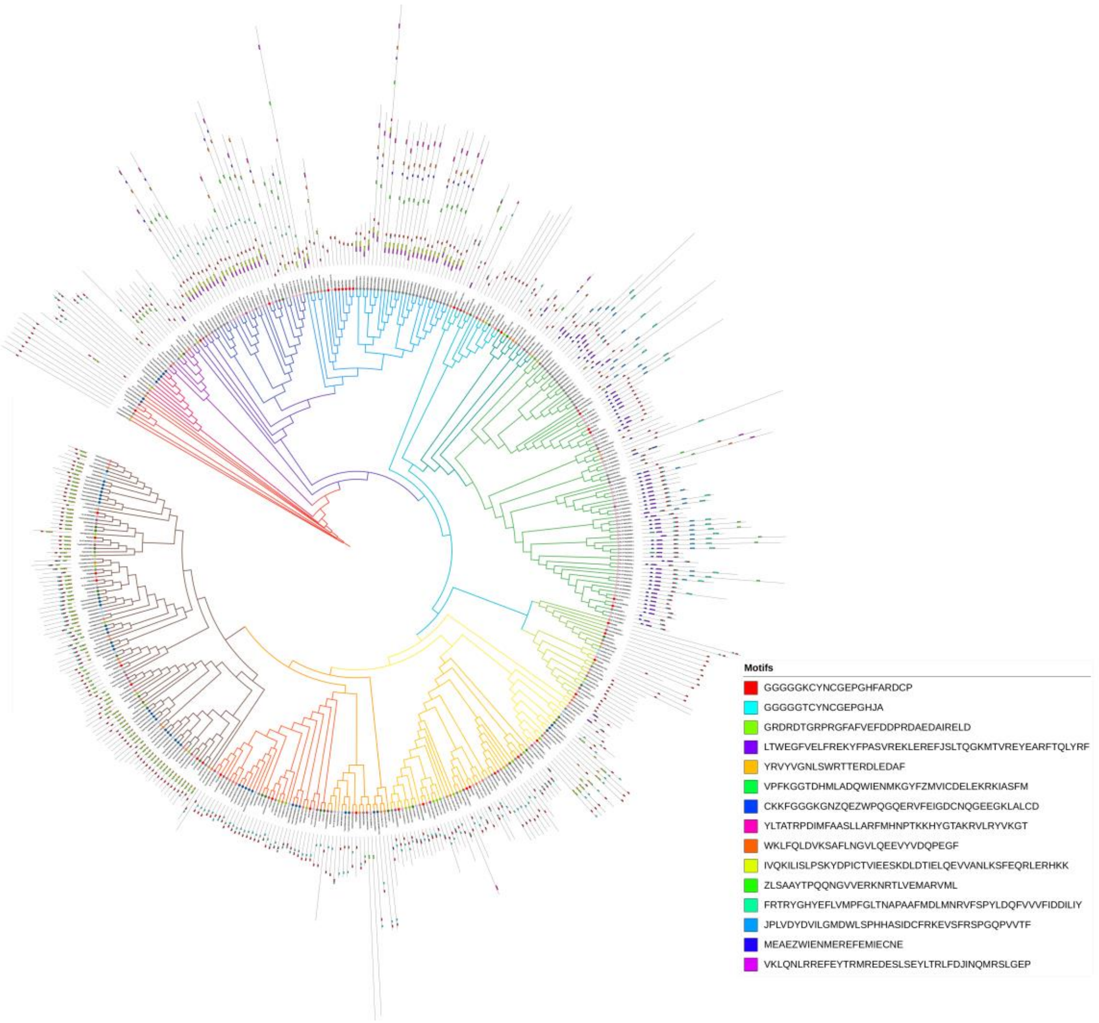

Figure S4 Phylogenetic tree of 450 *CCHC* genes. The colors of different branches and circles correspond to different subfamilies and species. The outermost layer represents the predicted motifs of CCHC proteins, while different colored squares representing different motifs.

2 Supporting Tables:

Table S1. Genome information for other 10 species

Common name	Scientific name	Chromosome number	Genome genes	*CCHC* genes
Ostreococcus	*Ostreococcus lucimarinus*	21	7603	4
Ginkgo	*Ginkgo biloba*	12	27,832	29
Rice	*Oryza sativa* subsp. *japonica*	12	42,582	26
Wheat	*Triticum aestivum*	21	133,346	50
Tomato	*Solanum lycopersicum*	12	30,389	31
Grape	*Vitis vinifera*	19	41,097	32
Arabidopsis	*Arabidopsis thaliana*	7	33,119	27
Strawberry	*Fragaria ananassa*	7	36,173	33
Peach	*Prunus persica*	8	35,389	89
Rose	*Rosa rugosa*	7	39,704	88

Table S2. 41 *CCHC* genes structure

Gene	Protein ID	Gene Range	Num of Exon	Num of Intron	Num of CDS[1]	Num of UTR[2]
RcCCHC1	PRQ55458	12545819:12548100	3	2	3	1
RcCCHC2	PRQ56287	26261196:26262440	3	2	3	1
RcCCHC3	PRQ56554	29541354:29542829	1	0	1	0
RcCCHC4	PRQ58706	53910596:53912048	2	1	2	2
RcCCHC5	PRQ58713	53982278:53987442	3	2	3	2
RcCCHC6	PRQ58731	54142965:54144107	1	0	1	2
RcCCHC7	PRQ58964	56019418:56022575	7	6	6	3
RcCCHC8	PRQ59045	56649101:56652599	4	3	4	2
RcCCHC9	PRQ59090	56929636:56932026	4	3	3	3
RcCCHC10	PRQ60098	64369354:64371753	5	4	5	2
RcCCHC11	PRQ60147	64662491:64666694	5	4	2	5
RcCCHC12	PRQ60191	64973569:64974473	2	1	2	2
RcCCHC13	PRQ60295	65656715:65659594	1	0	1	2
RcCCHC14	PRQ47404	11545027:11547438	2	1	1	3
RcCCHC15	PRQ51575	63657468:63661983	2	1	1	2
RcCCHC16	PRQ52325	71409913:71411711	3	2	3	2
RcCCHC17	PRQ42117	4294617:4297757	5	4	2	5
RcCCHC18	PRQ42351	6137075:6146026	1	0	1	2
RcCCHC19	PRQ42569	7601825:7607416	10	9	10	2
RcCCHC20	PRQ43392	14135149:14138047	7	6	6	3
RcCCHC21	PRQ45943	48923966:48929386	5	4	1	6

RcCCHC22	PRQ36998	14119818:14127422	4	3	2	4
RcCCHC23	PRQ39528	51757552:51764468	14	13	14	2
RcCCHC24	PRQ40949	63503252:63505785	3	2	2	3
RcCCHC25	PRQ41181	64963189:64966215	6	5	5	3
RcCCHC26	PRQ41372	66471804:66473990	5	4	5	2
RcCCHC27	PRQ29923	13563613:13566626	2	1	1	3
RcCCHC28	PRQ32678	46526590:46533672	4	3	1	5
RcCCHC29	PRQ34804	79227112:79229482	3	2	2	3
RcCCHC30	PRQ21890	405769:407352	1	0	1	2
RcCCHC31	PRQ22905	10702031:10707216	9	8	7	4
RcCCHC32	PRQ24003	24441141:24446020	4	3	3	3
RcCCHC33	PRQ26481	57199257:57200404	1	0	1	2
RcCCHC34	PRQ27425	64242703:64246043	6	5	5	3
RcCCHC35	PRQ16202	3218802:3219640	2	1	2	0
RcCCHC36	PRQ16817	7900882:7903501	3	2	3	2
RcCCHC37	PRQ17445	13019068:13021418	3	2	2	3
RcCCHC38	PRQ19099	30491276:30495175	3	2	3	1
RcCCHC39	PRQ21312	62969390:62973554	4	3	2	4
RcCCHC40	PRQ21314	62978520:62983394	5	4	2	5
RcCCHC41	PRQ21662	67751538:67752971	2	1	2	2

[1, 2] In this table, CDS and UTR represent coding sequence and untranslated region, respectively.

Table S3. The number of *CCHC* genes in different species.

Subfamily	Quantities	Algae	Gymnospermous	Amphibrya		Dicotyledon						
		Ostreococcus lucimarinus	***Ginkgo biloba***	***Oryza sativa* subsp. *japonica***	***Triticum aestivum***	***Solanum lycopersicum***	***Vitis vinifera***	***Arabidopsis thaliana***	***Fragaria ananassa***	***Prunus persica***	***Rosa chinensis***	***Rosa rugosa***
I	9	0	1	0	2	1	1	0	1	1	1	1
II	9	0	0	1	5	1	1	0	0	1	0	0
III	9	0	2	1	0	1	1	1	1	1	0	1
IV	7	0	0	0	0	1	1	0	1	2	1	1
V	29	0	0	1	0	0	0	1	2	8	1	16
VI	14	0	2	0	0	0	0	0	0	4	7	1
VII	27	0	0	0	0	0	0	0	0	27	0	0
VIII	16	1	3	0	0	2	1	3	2	1	2	1
IX	12	0	3	0	0	1	2	1	1	2	1	1
X	80	0	2	1	0	0	1	0	6	20	5	45
XI	10	1	0	1	0	1	1	1	1	1	2	1
XII	15	1	1	2	0	1	2	1	1	2	2	2
XIII	18	0	0	2	3	3	1	2	1	3	1	2
XIV	44	1	3	3	0	4	10	3	5	4	6	5
XV	18	0	2	2	4	2	0	1	2	2	1	2
XVI	34	0	1	2	8	4	3	4	4	3	3	2
XVII	99	0	9	10	28	9	7	9	5	7	8	7
total	450	4	29	26	50	31	32	27	33	89	41	88

Table S4. The Ka/Ks ratios and duplication gene pairs among rose and other species.

	geneID	gene 1	gene 2	Ka	Ks	Ka_Ks	Average Duplication time
Rosa chinensis & Fragaria ananassa	*RcCCHC4*	RchiOBHmChr1g0362231	FvesChr7G00343330.1	0.098534061	0.41762495	0.235939115	27.50443645
	RcCCHC5	RchiOBHmChr1g0362301	FvesChr7G00343440.1	0.044102441	0.190224051	0.231844713	
	RcCCHC6	RchiOBHmChr1g0362501	FvesChr7G00343660.1	0.052663584	0.296894165	0.177381673	
	RcCCHC7	RchiOBHmChr1g0364971	FvesChr7G00345890.1	0.118344662	0.330184573	0.358419719	
	RcCCHC8	RchiOBHmChr1g0365851	FvesChr7G00346700.1	0.077564255	0.217866362	0.356017582	
	RcCCHC9	RchiOBHmChr1g0366331	FvesChr7G00347280.1	0.027365829	0.369393006	0.074083234	
	RcCCHC10	RchiOBHmChr1g0377401	FvesChr7G00356310.1	0.047071141	0.238884088	0.197045946	
	RcCCHC13	RchiOBHmChr1g0379641	FvesChr7G00357650.1	0.115748579	0.251057589	0.461043934	
	RcCCHC14	RchiOBHmChr2g0099321	FvesChr1G00293250.1	0.056553144	0.528133136	0.107081227	
	RcCCHC14	RchiOBHmChr2g0099321	FvesChr2G00180170.1	0.287806763	0.870492092	0.330625362	
	RcCCHC16	RchiOBHmChr2g0154211	FvesChr6G00050930.1	0.194456489	0.409720233	0.474607972	
	RcCCHC19	RchiOBHmChr3g0459081	FvesChr6G00010410.1	0.024741439	0.195702846	0.1264235	
	RcCCHC20	RchiOBHmChr3g0468001	FvesChr7G00345890.1	0.356680139	NaN[1]	NaN	
	RcCCHC23	RchiOBHmChr4g0426211	FvesChr4G00157310.1	0.028253204	0.164741729	0.171499982	
	RcCCHC25	RchiOBHmChr4g0444111	FvesChr4G00173280.1	0.014793987	0.254209362	0.058196075	
	RcCCHC26	RchiOBHmChr4g0446201	FvesChr4G00175460.1	0.059359616	0.201392102	0.294746496	
	RcCCHC30	RchiOBHmChr6g0244251	FvesChr1G00293250.1	0.252354177	1.294132563	0.194998708	
	RcCCHC30	RchiOBHmChr6g0244251	FvesChr2G00180170.1	0.018587592	0.410195067	0.045314031	
	RcCCHC31	RchiOBHmChr6g0255381	FvesChr2G00184920.1	0.055205783	0.210340777	0.262458777	
	RcCCHC32	RchiOBHmChr6g0267601	FvesChr2G00191820.1	0.022294043	0.201771193	0.110491707	
	RcCCHC33	RchiOBHmChr6g0295081	FvesChr2G00212210.1	0.051663612	0.324778826	0.159073215	

	RcCCHC34	RchiOBHmChr6g0305171	FvesChr2G00223330.1	0.002397125	0.217218148	0.011035567	
	RcCCHC38	RchiOBHmChr7g0213381	FvesChr5G00258060.1	0.100379016	0.271311967	0.369976368	
	RcCCHC2	RchiOBHmChr1g0334111	rna-XM_062145718.1	0.109357342	0.236170354	0.463044323	
	RcCCHC4	RchiOBHmChr1g0362231	rna-XM_062140008.1	0.0107593	0.043290704	0.248536035	
	RcCCHC5	RchiOBHmChr1g0362301	rna-XM_062140014.1	0.001569449	0	/[2]	
	RcCCHC6	RchiOBHmChr1g0362501	rna-XM_062144294.1	0.008285088	0.057007488	0.145333336	
	RcCCHC7	RchiOBHmChr1g0364971	rna-XM_062141536.1	0.005756114	0.027727455	0.207596194	
	RcCCHC7	RchiOBHmChr1g0364971	rna-XM_062146079.1	0.202234847	2.046396549	0.098824857	
	RcCCHC8	RchiOBHmChr1g0365851	rna-XM_062144364.1	0.016438148	0.045974632	0.357548229	
	RcCCHC9	RchiOBHmChr1g0366331	rna-XM_062144769.1	0.005495369	0.105147281	0.052263537	
	RcCCHC14	RchiOBHmChr2g0099321	rna-XM_062143369.1	0.003442347	0.064325116	0.053514817	
	RcCCHC14	RchiOBHmChr2g0099321	rna-XM_062167866.1	0.243810847	1.062905216	0.229381552	
	RcCCHC16	RchiOBHmChr2g0154211	rna-XM_062153185.1	0.016241236	0.038647004	0.420245675	
Rosa chinensis & Rosa rugosa	*RcCCHC17*	RchiOBHmChr3g0454141	rna-XM_062147046.1	0.00197596	0.05549526	0.035605929	24.76272556
	RcCCHC19	RchiOBHmChr3g0459081	rna-XM_062147225.1	0.004710978	0.032061872	0.146933975	
	RcCCHC20	RchiOBHmChr3g0468001	rna-XM_062141536.1	0.190290176	2.397728242	0.079362695	
	RcCCHC20	RchiOBHmChr3g0468001	rna-XM_062146079.1	0.001599574	0.056485196	0.028318465	
	RcCCHC22	RchiOBHmChr4g0397711	rna-XM_062139024.1	0.06055866	0.112995206	0.535940084	
	RcCCHC23	RchiOBHmChr4g0426211	rna-XM_062138678.1	0.00572067	0.016002385	0.357488569	
	RcCCHC25	RchiOBHmChr4g0444111	rna-XM_062136246.1	0.008601364	0.066749701	0.128859963	
	RcCCHC26	RchiOBHmChr4g0446201	rna-XM_062134126.1	0.009038523	0.068288609	0.132357692	
	RcCCHC28	RchiOBHmChr5g0049101	rna-XM_062158213.1	0.047825952	0.07760965	0.616237175	
	RcCCHC30	RchiOBHmChr6g0244251	rna-XM_062143369.1	0.229297607	1.213865811	0.188898645	
	RcCCHC30	RchiOBHmChr6g0244251	rna-XM_062167866.1	0	0.100596975	0	
	RcCCHC32	RchiOBHmChr6g0267601	rna-XM_062165867.1	0.001648352	0.022729012	0.072521951	
	RcCCHC34	RchiOBHmChr6g0305171	rna-XM_062162705.1	0	0.020919427	0	

	RcCCHC38	RchiOBHmChr7g0213381	rna-XM_062169816.1	0.034807517	0.078766662	0.44190671	
	RcCCHC5	RchiOBHmChr1g0362301	Pp02G027490.1	0.03335308	0.418533522	0.079690343	
	RcCCHC6	RchiOBHmChr1g0362501	Pp02G027630.1	0.14056047	0.484643747	0.290028441	
	RcCCHC7	RchiOBHmChr1g0364971	Pp02G029040.1	0.051655522	0.473994568	0.108979143	
	RcCCHC7	RchiOBHmChr1g0364971	Pp06G011390.1	0.262489467	2.066252268	0.127036505	
	RcCCHC8	RchiOBHmChr1g0365851	Pp02G029560.2	0.197910638	0.562001778	0.352153046	
	RcCCHC10	RchiOBHmChr1g0377401	Pp02G035570.1	0.15682189	0.496300681	0.315981613	
	RcCCHC14	RchiOBHmChr2g0099321	Pp07G009660.1	0.101088094	1.2854503	0.078640219	
	RcCCHC14	RchiOBHmChr2g0099321	Pp08G019570.1	0.246142371	1.401407735	0.17563937	
	RcCCHC16	RchiOBHmChr2g0154211	Pp03G024440.1	0.182386936	0.511283262	0.356723855	
Rosa chinensis & Prunus persica	*RcCCHC17*	RchiOBHmChr3g0454141	Pp06G003350.1	0.026035672	0.486685444	0.053495892	59.8588637
	RcCCHC19	RchiOBHmChr3g0459081	Pp06G005850.1	0.085607289	0.469514691	0.182331438	
	RcCCHC20	RchiOBHmChr3g0468001	Pp02G029040.1	0.196654724	1.795350826	0.109535541	
	RcCCHC20	RchiOBHmChr3g0468001	Pp06G011390.1	0.118955494	0.566342202	0.210041726	
	RcCCHC23	RchiOBHmChr4g0426211	Pp01G011560.1	0.078639437	0.344570913	0.228224246	
	RcCCHC25	RchiOBHmChr4g0444111	Pp01G032410.1	0.063539245	0.572092487	0.111064637	
	RcCCHC26	RchiOBHmChr4g0446201	Pp01G041250.1	0.099758546	0.407867656	0.244585578	
	RcCCHC30	RchiOBHmChr6g0244251	Pp07G009660.1	0.273573305	1.607842122	0.170149358	
	RcCCHC30	RchiOBHmChr6g0244251	Pp08G019570.1	0.119143719	1.15543861	0.103115577	
	RcCCHC32	RchiOBHmChr6g0267601	Pp08G012040.1	0.029736303	0.388469737	0.076547284	
	RcCCHC33	RchiOBHmChr6g0295081	Pp01G056450.1	0.112763267	0.590510683	0.190958894	
	RcCCHC34	RchiOBHmChr6g0305171	Pp01G051830.1	0.027629746	0.568560895	0.048595932	
	RcCCHC38	RchiOBHmChr7g0213381	Pp05G023370.1	0.242797261	0.46652089	0.520442421	
Rosa chinensis & Arabidopsis	*RcCCHC5*	RchiOBHmChr1g0362301	AT5G04280.1	0.240254617	1.54248197	0.155758461	146.1694473
	RcCCHC6	RchiOBHmChr1g0362501	AT3G10400.1	0.24945401	1.833632002	0.136043661	
	RcCCHC8	RchiOBHmChr1g0365851	AT3G55340.1	0.425226663	1.889902863	0.224999216	

thaliana	*RcCCHC10*	RchiOBHmChr1g0377401	AT5G52380.1	0.288204922	2.328553368	0.123769945	
	RcCCHC14	RchiOBHmChr2g0099321	AT2G17870.1	0.320148102	NaN	NaN	
	RcCCHC14	RchiOBHmChr2g0099321	AT4G36020.1	0.332756346	NaN	NaN	
	RcCCHC19	RchiOBHmChr3g0459081	AT5G26742.2	0.205626265	1.486212782	0.138355872	
	RcCCHC20	RchiOBHmChr3g0468001	AT2G37340.1	0.205247309	2.478410792	0.08281408	
	RcCCHC20	RchiOBHmChr3g0468001	AT3G53500.2	0.257587555	2.217637865	0.116154021	
	RcCCHC25	RchiOBHmChr4g0444111	AT1G23860.1	0.161178845	2.115851927	0.076176808	
	RcCCHC30	RchiOBHmChr6g0244251	AT2G21060.1	0.17856055	1.708613301	0.104506122	
	RcCCHC30	RchiOBHmChr6g0244251	AT4G38680.1	0.203762359	1.532546232	0.132956745	
	RcCCHC34	RchiOBHmChr6g0305171	AT2G24590.1	0.134778409	1.512258736	0.089123908	
	RcCCHC34	RchiOBHmChr6g0305171	AT4G31580.1	0.126523997	2.29027261	0.055244077	
	RcCCHC38	RchiOBHmChr7g0213381	AT4G19130.2	0.380567713	2.079914877	0.182972735	
	RcCCHC38	RchiOBHmChr7g0213381	AT5G45400.1	0.367963724	1.586550087	0.231926951	
Rosa chinensis & Solanum lycopersicum	*RcCCHC5*	RchiOBHmChr1g0362301	SlT11G003060.1	0.211589082	1.471510167	0.143790431	148.9101261
	RcCCHC6	RchiOBHmChr1g0362501	SlT11G003160.1	0.279602996	NaN	NaN	
	RcCCHC7	RchiOBHmChr1g0364971	SlT05G022370.1	0.17974237	1.418125548	0.126746444	
	RcCCHC7	RchiOBHmChr1g0364971	SlT09G001010.1	0.304749348	2.653660264	0.114841132	
	RcCCHC14	RchiOBHmChr2g0099321	SlT01G039460.1	0.312600545	2.815439677	0.111030809	
	RcCCHC14	RchiOBHmChr2g0099321	SlT02G022230.1	0.294199765	2.797951838	0.105148259	
	RcCCHC14	RchiOBHmChr2g0099321	SlT03G005810.1	0.288179866	2.050146965	0.140565467	
	RcCCHC20	RchiOBHmChr3g0468001	SlT05G022370.1	0.200061029	1.959874097	0.102078511	
	RcCCHC20	RchiOBHmChr3g0468001	SlT09G001010.1	0.286669072	1.254706548	0.228474995	
	RcCCHC23	RchiOBHmChr4g0426211	SlT01G012930.1	0.195910192	1.428226008	0.137170302	
	RcCCHC26	RchiOBHmChr4g0446201	SlT04G002770.1	0.361326705	1.858703194	0.194397204	
	RcCCHC30	RchiOBHmChr6g0244251	SlT01G039460.1	0.262603533	NaN	NaN	
	RcCCHC30	RchiOBHmChr6g0244251	SlT10G015720.1	0.217800592	NaN	NaN	

	RcCCHC32	RchiOBHmChr6g0267601	SlT10G014930.1	0.100118253	1.635206264	0.061226681	
	RcCCHC34	RchiOBHmChr6g0305171	SlT08G015510.1	0.105896607	2.075276517	0.051027709	
	RcCCHC38	RchiOBHmChr7g0213381	SlT08G017190.1	0.342638179	1.746984221	0.196131239	
**Rosa chinensis & Vitis vinifera**	*RcCCHC5*	RchiOBHmChr1g0362301	Vitvi13g00684_t001	0.112507668	0.929592005	0.121029083	89.49253377
	RcCCHC5	RchiOBHmChr1g0362301	Vitvi08g01612_t001	0.204812133	1.457404487	0.140532114	
	RcCCHC6	RchiOBHmChr1g0362501	Vitvi13g00697_t001	0.291601753	0.887975443	0.328389434	
	RcCCHC7	RchiOBHmChr1g0364971	Vitvi13g00214_t001	0.107715498	1.339302423	0.080426569	
	RcCCHC7	RchiOBHmChr1g0364971	Vitvi08g01113_t001	0.190821209	1.205146688	0.158338575	
	RcCCHC8	RchiOBHmChr1g0365851	Vitvi13g00259_t001	0.253736642	1.022003112	0.248273845	
	RcCCHC9	RchiOBHmChr1g0366331	Vitvi13g00274_t001	0.10560227	1.116721851	0.094564524	
	RcCCHC10	RchiOBHmChr1g0377401	Vitvi16g01027_t001	0.287916721	1.170513692	0.245974671	
	RcCCHC14	RchiOBHmChr2g0099321	Vitvi03g00121_t001	0.270688995	1.636095528	0.16544816	
	RcCCHC14	RchiOBHmChr2g0099321	Vitvi04g01469_t001	0.213659782	1.694224825	0.126110643	
	RcCCHC19	RchiOBHmChr3g0459081	Vitvi15g04674_t001	0.157045116	0.873756263	0.179735611	
	RcCCHC20	RchiOBHmChr3g0468001	Vitvi13g00214_t001	0.19053893	1.641413622	0.116082215	
	RcCCHC20	RchiOBHmChr3g0468001	Vitvi08g01113_t001	0.169177656	1.360803878	0.12432185	
	RcCCHC23	RchiOBHmChr4g0426211	Vitvi05g00168_t001	0.137893832	0.799051016	0.172572	
	RcCCHC23	RchiOBHmChr4g0426211	Vitvi05g04051_t001	0.214756649	1.004555513	0.213782758	
	RcCCHC25	RchiOBHmChr4g0444111	Vitvi01g02080_t001	0.112995206	0.694580893	0.162681132	
	RcCCHC26	RchiOBHmChr4g0446201	Vitvi01g01435_t001	0.23746354	1.08747293	0.218362714	
	RcCCHC30	RchiOBHmChr6g0244251	Vitvi03g00121_t001	0.170412658	1.164397335	0.146352669	
	RcCCHC32	RchiOBHmChr6g0267601	Vitvi03g00043_t002	0.08731047	0.921716431	0.094725956	
	RcCCHC33	RchiOBHmChr6g0295081	Vitvi18g00309_t001	0.238064685	1.478521659	0.161015351	
	RcCCHC34	RchiOBHmChr6g0305171	Vitvi04g04188_t003	0.061930717	0.876955234	0.070620158	
	RcCCHC38	RchiOBHmChr7g0213381	Vitvi02g01153_t001	0.27646421	1.232659831	0.224282647	
Rosa	*RcCCHC8*	RchiOBHmChr1g0365851	Os05t0114500-00	0.513614067	2.293069382	0.223985402	156.605879

chinensis & Oryza sativa* subsp. *japonica	*RcCCHC20*	RchiOBHmChr3g0468001	Os05t0120100-01	0.279334099	1.778683473	0.157045423	
Rosa chinensis & Ginkgo biloba	*RcCCHC14*	RchiOBHmChr2g0099321	chr6.933	0.302995365	2.931011238	0.103375709	197.2993241
	RcCCHC30	RchiOBHmChr6g0244251	chr4.2341	0.241752923	2.73423804	0.088416926	
	RcCCHC33	RchiOBHmChr6g0295081	chr6.933	0.291299353	2.661863471	0.10943437	
	RcCCHC25	RchiOBHmChr4g0444111	chr2.105	0.151541178	1.932452102	0.078419112	

[1] The “Na” symbol indicates that the divergence of the gene pair is too significant and the evolutionary distance is considerable, rendering calculation impossible.

[2] The symbol “/” indicates that the Ks value of the gene pair is 0, and the Ka/Ks ratio cannot be calculated.

Table S5. Information for cis-acting elements of *RcCCHC* genes.

Gene ID	Cis-acting elements	Motifs	Information sources
RcCCHC1	ARE	AAACCA	*Zea mays*
RcCCHC1	as-1	TGACG	*Arabidopsis thaliana*
RcCCHC1	CAT-box	GCCACT	*Arabidopsis thaliana*
RcCCHC1	CGTCA-motif	CGTCA	*Hordeum vulgare*
RcCCHC1	circadian	CAAAGATATC	*Lycopersicon esculentum*
RcCCHC1	DRE core	GCCGAC	*Arabidopsis thaliana*
RcCCHC1	ERE	ATTTCATA	*Nicotiana glutinos*
RcCCHC1	G-box	CACGAC	*Zea mays*
RcCCHC1	GT1-motif	GGTTAA	*Arabidopsis thaliana*
RcCCHC1	I-box	TGATAATGT	*Solanum tuberosum*
RcCCHC1	MRE	AACCTAA	*Petroselinum crispum*
RcCCHC1	MYB	CAACCA	*Arabidopsis thaliana*
RcCCHC1	MYC	CATTTG	*Arabidopsis thaliana*
RcCCHC1	STRE	AGGGG	*Arabidopsis thaliana*
RcCCHC1	TATC-box	TATCCCA	*Oryza sativa*
RcCCHC1	TCA-element	TCAGAAGAGG	*Brassica oleracea*
RcCCHC1	TGACG-motif	TGACG	*Hordeum vulgare*
RcCCHC1	W box	TTGACC	*Arabidopsis thaliana*
RcCCHC2	ARE	AAACCA	*Zea mays*
RcCCHC2	as-1	TGACG	*Arabidopsis thaliana*
RcCCHC2	Box 4	ATTAAT	*Petroselinum crispum*
RcCCHC2	CGTCA-motif	CGTCA	*Hordeum vulgare*
RcCCHC2	GATA-motif	AAGGATAAGG	*Solanum tuberosum*
RcCCHC2	G-box	CACGAC	*Zea mays*
RcCCHC2	MBS	CAACTG	*Arabidopsis thaliana*
RcCCHC2	MYB	TAACCA	*Arabidopsis thaliana*
RcCCHC2	MYC	CAATTG	*Arabidopsis thaliana*
RcCCHC2	P-box	CCTTTTG	*Oryza sativa*
RcCCHC2	TCA-element	CCATCTTTTT	*Nicotiana tabacum*
RcCCHC2	TC-rich repeats	GTTTTCTTAC	*Nicotiana tabacum*
RcCCHC2	TGACG-motif	TGACG	*Hordeum vulgare*
RcCCHC2	TGA-element	AACGAC	*Brassica oleracea*
RcCCHC3	ABRE	GCCGCGTGGC	*Oryza sativa*
RcCCHC3	ARE	AAACCA	*Zea mays*
RcCCHC3	as-1	TGACG	*Arabidopsis thaliana*
RcCCHC3	Box 4	ATTAAT	*Petroselinum crispum*
RcCCHC3	CAT-box	GCCACT	*Arabidopsis thaliana*
RcCCHC3	CGTCA-motif	CGTCA	*Hordeum vulgare*
RcCCHC3	G-box	ACACGTGT	*Brassica napus*
RcCCHC3	GT1-motif	GGTTAA	*Arabidopsis thaliana*
RcCCHC3	LTR	CCGAAA	*Hordeum vulgare*
RcCCHC3	MBS	CAACTG	*Arabidopsis thaliana*

RcCCHC3	MYB	CAACAG	*Arabidopsis thaliana*
RcCCHC3	MYC	CATTTG	*Arabidopsis thaliana*
RcCCHC3	STRE	AGGGG	*Arabidopsis thaliana*
RcCCHC3	STRE	AGGGG	*Arabidopsis thaliana*
RcCCHC3	TGACG-motif	TGACG	*Hordeum vulgare*
RcCCHC3	TGA-element	AACGAC	*Brassica oleracea*
RcCCHC3	W box	TTGACC	*Arabidopsis thaliana*
RcCCHC4	ABRE	ACGTG	*Arabidopsis thaliana*
RcCCHC4	ARE	AAACCA	*Zea mays*
RcCCHC4	as-1	TGACG	*Arabidopsis thaliana*
RcCCHC4	Box 4	ATTAAT	*Petroselinum crispum*
RcCCHC4	CGTCA-motif	CGTCA	*Hordeum vulgare*
RcCCHC4	ERE	ATTTTAAA	*Nicotiana glutinos*
RcCCHC4	GA-motif	ATAGATAA	*Arabidopsis thaliana*
RcCCHC4	G-Box	CACGTT	*Pisum sativum*
RcCCHC4	GT1-motif	GGTTAA	*Arabidopsis thaliana*
RcCCHC4	MBS	CAACTG	*Arabidopsis thaliana*
RcCCHC4	MRE	AACCTAA	*Petroselinum crispum*
RcCCHC4	MYB	CAACCA	*Arabidopsis thaliana*
RcCCHC4	MYC	CATTTG	*Arabidopsis thaliana*
RcCCHC4	P-box	CCTTTTG	*Oryza sativa*
RcCCHC4	STRE	AGGGG	*Arabidopsis thaliana*
RcCCHC4	TCA-element	CCATCTTTTT	*Nicotiana tabacum*
RcCCHC4	TGACG-motif	TGACG	*Hordeum vulgare*
RcCCHC4	WUN-motif	AAATTACT	*Nicotiana glutinosa*
RcCCHC5	ARE	AAACCA	*Zea mays*
RcCCHC5	as-1	TGACG	*Arabidopsis thaliana*
RcCCHC5	CGTCA-motif	CGTCA	*Hordeum vulgare*
RcCCHC5	LTR	CCGAAA	*Hordeum vulgare*
RcCCHC5	MBS	CAACTG	*Arabidopsis thaliana*
RcCCHC5	MYB	TAACCA	*Arabidopsis thaliana*
RcCCHC5	MYC	CATGTG	*Arabidopsis thaliana*
RcCCHC5	P-box	CCTTTTG	*Oryza sativa*
RcCCHC5	STRE	AGGGG	*Arabidopsis thaliana*
RcCCHC5	TATC-box	TATCCCA	*Oryza sativa*
RcCCHC5	TCA-element	CCATCTTTTT	*Nicotiana tabacum*
RcCCHC5	TGACG-motif	TGACG	*Hordeum vulgare*
RcCCHC5	TGA-element	AACGAC	*Brassica oleracea*
RcCCHC5	W box	TTGACC	*Arabidopsis thaliana*
RcCCHC5	WUN-motif	CCATTTCAA	*Nicotiana glutinosa*
RcCCHC6	ABRE	CACGTG	*Arabidopsis thaliana*
RcCCHC6	ARE	AAACCA	*Zea mays*
RcCCHC6	as-1	TGACG	*Arabidopsis thaliana*
RcCCHC6	CAT-box	GCCACT	*Arabidopsis thaliana*

RcCCHC6	CGTCA-motif	CGTCA	*Hordeum vulgare*
RcCCHC6	GATA-motif	AAGATAAGATT	*Arabidopsis thaliana*
RcCCHC6	G-Box	CACGTG	*Pisum sativum*
RcCCHC6	GT1-motif	GGTTAA	*Arabidopsis thaliana*
RcCCHC6	MBS	CAACTG	*Arabidopsis thaliana*
RcCCHC6	MYB	CAACAG	*Arabidopsis thaliana*
RcCCHC6	MYC	CATGTG	*Arabidopsis thaliana*
RcCCHC6	P-box	CCTTTTG	*Oryza sativa*
RcCCHC6	STRE	AGGGG	*Arabidopsis thaliana*
RcCCHC6	TGACG-motif	TGACG	*Hordeum vulgare*
RcCCHC6	TGA-element	AACGAC	*Brassica oleracea*
RcCCHC6	W box	TTGACC	*Arabidopsis thaliana*
RcCCHC7	A-box	CCGTCC	*Petroselinum crispum*
RcCCHC7	ARE	AAACCA	*Zea mays*
RcCCHC7	as-1	TGACG	*Arabidopsis thaliana*
RcCCHC7	Box 4	ATTAAT	*Petroselinum crispum*
RcCCHC7	CGTCA-motif	CGTCA	*Hordeum vulgare*
RcCCHC7	GT1-motif	GGTTAAT	*Avena sativa*
RcCCHC7	MYB	TAACCA	*Arabidopsis thaliana*
RcCCHC7	MYC	CATGTG	*Arabidopsis thaliana*
RcCCHC7	P-box	CCTTTTG	*Oryza sativa*
RcCCHC7	STRE	AGGGG	*Arabidopsis thaliana*
RcCCHC7	TCA-element	CCATCTTTTT	*Nicotiana tabacum*
RcCCHC7	TGACG-motif	TGACG	*Hordeum vulgare*
RcCCHC7	WUN-motif	AAATTTCTT	*Nicotiana glutinosa*
RcCCHC8	ABRE	CACGTG	*Arabidopsis thaliana*
RcCCHC8	ARE	AAACCA	*Zea mays*
RcCCHC8	Box 4	ATTAAT	*Petroselinum crispum*
RcCCHC8	circadian	CAAAGATATC	*Lycopersicon esculentum*
RcCCHC8	GATA-motif	AAGGATAAGG	*Solanum tuberosum*
RcCCHC8	G-Box	CACGTG	*Pisum sativum*
RcCCHC8	GT1-motif	GGTTAA	*Arabidopsis thaliana*
RcCCHC8	I-box	AGATAAGG	*Triticum aestivum*
RcCCHC8	MBS	CAACTG	*Arabidopsis thaliana*
RcCCHC8	MYB	TAACCA	*Arabidopsis thaliana*
RcCCHC8	MYC	CATTTG	*Arabidopsis thaliana*
RcCCHC8	TGA-element	AACGAC	*Brassica oleracea*
RcCCHC9	ABRE	GACACGTGGC	*Triticum aestivum*
RcCCHC9	as-1	TGACG	*Arabidopsis thaliana*
RcCCHC9	CGTCA-motif	CGTCA	*Hordeum vulgare*
RcCCHC9	GATA-motif	GATAGGG	*Pisum sativum*
RcCCHC9	G-box	TACGTG	*Arabidopsis thaliana*
RcCCHC9	GT1-motif	GGTTAAT	*Avena sativa*
RcCCHC9	MYB	CAACAG	*Arabidopsis thaliana*

RcCCHC9	MYC	CATTTG	*Arabidopsis thaliana*
RcCCHC9	STRE	AGGGG	*Arabidopsis thaliana*
RcCCHC9	TATC-box	TATCCCA	*Oryza sativa*
RcCCHC9	TGACG-motif	TGACG	*Hordeum vulgare*
RcCCHC9	TGA-element	AACGAC	*Brassica oleracea*
RcCCHC9	W box	TTGACC	*Arabidopsis thaliana*
RcCCHC10	ABRE	ACGTG	*Arabidopsis thaliana*
RcCCHC10	as-1	TGACG	*Arabidopsis thaliana*
RcCCHC10	Box 4	ATTAAT	*Petroselinum crispum*
RcCCHC10	CGTCA-motif	CGTCA	*Hordeum vulgare*
RcCCHC10	circadian	CAAAGATATC	*Lycopersicon esculentum*
RcCCHC10	ERE	ATTTCATA	*Nicotiana glutinos*
RcCCHC10	GATA-motif	GATAGGA	*Arabidopsis thaliana*
RcCCHC10	G-box	TACGTG	*Arabidopsis thaliana*
RcCCHC10	GT1-motif	GGTTAA	*Arabidopsis thaliana*
RcCCHC10	MYC	CATGTG	*Arabidopsis thaliana*
RcCCHC10	STRE	AGGGG	*Arabidopsis thaliana*
RcCCHC10	TGACG-motif	TGACG	*Hordeum vulgare*
RcCCHC10	WUN-motif	CCATTTCAA	*Nicotiana glutinosa*
RcCCHC11	ARE	AAACCA	*Zea mays*
RcCCHC11	as-1	TGACG	*Arabidopsis thaliana*
RcCCHC11	CGTCA-motif	CGTCA	*Hordeum vulgare*
RcCCHC11	G-box	CACGAC	*Zea mays*
RcCCHC11	GT1-motif	GGTTAA	*Arabidopsis thaliana*
RcCCHC11	LTR	CCGAAA	*Hordeum vulgare*
RcCCHC11	MYC	CATTTG	*Arabidopsis thaliana*
RcCCHC11	P-box	CCTTTTG	*Oryza sativa*
RcCCHC11	STRE	AGGGG	*Arabidopsis thaliana*
RcCCHC11	TCA-element	TCAGAAGAGG	*Brassica oleracea*
RcCCHC11	TGACG-motif	TGACG	*Hordeum vulgare*
RcCCHC12	ARE	AAACCA	*Zea mays*
RcCCHC12	GT1-motif	GGTTAA	*Arabidopsis thaliana*
RcCCHC12	I-box	cCATATCCAAT	*Flaveria trinervia*
RcCCHC12	MBS	CAACTG	*Arabidopsis thaliana*
RcCCHC12	MRE	AACCTAA	*Petroselinum crispum*
RcCCHC12	MYB	TAACCA	*Arabidopsis thaliana*
RcCCHC12	MYC	CAATTG	*Arabidopsis thaliana*
RcCCHC12	RY-element	CATGCATG	*Helianthus annuus*
RcCCHC12	TC-rich repeats	GTTTTCTTAC	*Nicotiana tabacum*
RcCCHC12	W box	TTGACC	*Arabidopsis thaliana*
RcCCHC12	WUN-motif	AAATTTCTT	*Nicotiana glutinosa*
RcCCHC13	ARE	AAACCA	*Zea mays*
RcCCHC13	as-1	TGACG	*Arabidopsis thaliana*
RcCCHC13	Box 4	ATTAAT	*Petroselinum crispum*

RcCCHC13	GATA-motif	AAGGATAAGG	*Solanum tuberosum*
RcCCHC13	G-box	CACGAC	*Zea mays*
RcCCHC13	GT1-motif	GGTTAA	*Arabidopsis thaliana*
RcCCHC13	I-box	gGATAAGGTG	*Zea mays*
RcCCHC13	LTR	CCGAAA	*Hordeum vulgare*
RcCCHC13	MBS	CAACTG	*Arabidopsis thaliana*
RcCCHC13	MYB	CAACCA	*Arabidopsis thaliana*
RcCCHC13	MYC	CAATTG	*Arabidopsis thaliana*
RcCCHC13	P-box	CCTTTTG	*Oryza sativa*
RcCCHC13	STRE	AGGGG	*Arabidopsis thaliana*
RcCCHC13	TCA-element	CCATCTTTTT	*Nicotiana tabacum*
RcCCHC13	TC-rich repeats	ATTCTCTAAC	*Nicotiana tabacum*
RcCCHC13	TGACG-motif	TGACG	*Hordeum vulgare*
RcCCHC13	W box	TTGACC	*Arabidopsis thaliana*
RcCCHC14	ARE	AAACCA	*Zea mays*
RcCCHC14	as-1	TGACG	*Arabidopsis thaliana*
RcCCHC14	Box 4	ATTAAT	*Petroselinum crispum*
RcCCHC14	CGTCA-motif	CGTCA	*Hordeum vulgare*
RcCCHC14	ERE	ATTTCATA	*Nicotiana glutinos*
RcCCHC14	G-Box	TCCACATGGCA	*Triticum aestivum*
RcCCHC14	GT1-motif	GGTTAA	*Arabidopsis thaliana*
RcCCHC14	MBS	CAACTG	*Arabidopsis thaliana*
RcCCHC14	MYB	TAACCA	*Arabidopsis thaliana*
RcCCHC14	MYC	CATTTG	*Arabidopsis thaliana*
RcCCHC14	P-box	CCTTTTG	*Oryza sativa*
RcCCHC14	STRE	AGGGG	*Arabidopsis thaliana*
RcCCHC14	TGACG-motif	TGACG	*Hordeum vulgare*
RcCCHC14	TGA-element	AACGAC	*Brassica oleracea*
RcCCHC15	A-box	CCGTCC	*Petroselinum crispum*
RcCCHC15	ABRE	ACGTG	*Arabidopsis thaliana*
RcCCHC15	ARE	AAACCA	*Zea mays*
RcCCHC15	as-1	TGACG	*Arabidopsis thaliana*
RcCCHC15	Box 4	ATTAAT	*Petroselinum crispum*
RcCCHC15	CAT-box	GCCACT	*Arabidopsis thaliana*
RcCCHC15	CGTCA-motif	CGTCA	*Hordeum vulgare*
RcCCHC15	DRE core	GCCGAC	*Arabidopsis thaliana*
RcCCHC15	ERE	ATTTCATA	*Nicotiana glutinos*
RcCCHC15	G-box	CACGTC	*Zea mays*
RcCCHC15	GCN4_motif	TGAGTCA	*Oryza sativa*
RcCCHC15	LTR	CCGAAA	*Hordeum vulgare*
RcCCHC15	MBS	CAACTG	*Arabidopsis thaliana*
RcCCHC15	MYB	TAACCA	*Arabidopsis thaliana*
RcCCHC15	MYC	CATTTG	*Arabidopsis thaliana*
RcCCHC15	STRE	AGGGG	*Arabidopsis thaliana*

RcCCHC15	TATC-box	TATCCCA	*Oryza sativa*
RcCCHC15	TCA-element	CCATCTTTTT	*Nicotiana tabacum*
RcCCHC15	TGACG-motif	TGACG	*Hordeum vulgare*
RcCCHC15	W box	TTGACC	*Arabidopsis thaliana*
RcCCHC15	WUN-motif	AAATTTCCT	*Brassica oleracea*
RcCCHC16	ABRE	ACGTG	*Arabidopsis thaliana*
RcCCHC16	ARE	AAACCA	*Zea mays*
RcCCHC16	as-1	TGACG	*Arabidopsis thaliana*
RcCCHC16	Box 4	ATTAAT	*Petroselinum crispum*
RcCCHC16	CAT-box	GCCACT	*Arabidopsis thaliana*
RcCCHC16	CGTCA-motif	CGTCA	*Hordeum vulgare*
RcCCHC16	ERE	ATTTTAAA	*Nicotiana glutinos*
RcCCHC16	G-box	CACGAC	*Zea mays*
RcCCHC16	GCN4_motif	TGAGTCA	*Oryza sativa*
RcCCHC16	GT1-motif	GGTTAA	*Arabidopsis thaliana*
RcCCHC16	MRE	AACCTAA	*Petroselinum crispum*
RcCCHC16	MYB	TAACCA	*Arabidopsis thaliana*
RcCCHC16	MYC	CATGTG	*Arabidopsis thaliana*
RcCCHC16	STRE	AGGGG	*Arabidopsis thaliana*
RcCCHC16	TGACG-motif	TGACG	*Hordeum vulgare*
RcCCHC17	ABRE	ACGTG	*Arabidopsis thaliana*
RcCCHC17	ARE	AAACCA	*Zea mays*
RcCCHC17	as-1	TGACG	*Arabidopsis thaliana*
RcCCHC17	CGTCA-motif	CGTCA	*Hordeum vulgare*
RcCCHC17	GA-motif	ATAGATAA	*Arabidopsis thaliana*
RcCCHC17	G-Box	CACGTG	*Pisum sativum*
RcCCHC17	GT1-motif	GGTTAA	*Arabidopsis thaliana*
RcCCHC17	MYB	CAACAG	*Arabidopsis thaliana*
RcCCHC17	MYC	CAATTG	*Arabidopsis thaliana*
RcCCHC17	STRE	AGGGG	*Arabidopsis thaliana*
RcCCHC17	TGACG-motif	TGACG	*Hordeum vulgare*
RcCCHC17	TGA-element	AACGAC	*Brassica oleracea*
RcCCHC17	W box	TTGACC	*Arabidopsis thaliana*
RcCCHC17	WUN-motif	AAATTACTA	*Nicotiana glutinosa*
RcCCHC18	ARE	AAACCA	*Zea mays*
RcCCHC18	Box 4	ATTAAT	*Petroselinum crispum*
RcCCHC18	CAT-box	GCCACT	*Arabidopsis thaliana*
RcCCHC18	circadian	CAAAGATATC	*Lycopersicon esculentum*
RcCCHC18	DRE core	GCCGAC	*Arabidopsis thaliana*
RcCCHC18	ERE	ATTTCATA	*Nicotiana glutinos*
RcCCHC18	G-box	CAGACGTGGCA	*Nicotiana plumbaginifolia*
RcCCHC18	GT1-motif	GGTTAA	*Arabidopsis thaliana*
RcCCHC18	MRE	AACCTAA	*Petroselinum crispum*
RcCCHC18	MYB	CAACCA	*Arabidopsis thaliana*

RcCCHC18	MYC	CATTTG	*Arabidopsis thaliana*
RcCCHC18	STRE	AGGGG	*Arabidopsis thaliana*
RcCCHC18	TGA-element	AACGAC	*Brassica oleracea*
RcCCHC18	W box	TTGACC	*Arabidopsis thaliana*
RcCCHC19	ABRE	ACGTG	*Arabidopsis thaliana*
RcCCHC19	Box 4	ATTAAT	*Petroselinum crispum*
RcCCHC19	CAT-box	GCCACT	*Arabidopsis thaliana*
RcCCHC19	ERE	ATTTTAAA	*Nicotiana glutinos*
RcCCHC19	GATA-motif	GATAGGA	*Arabidopsis thaliana*
RcCCHC19	G-box	CACGTC	*Zea mays*
RcCCHC19	LTR	CCGAAA	*Hordeum vulgare*
RcCCHC19	MBS	CAACTG	*Arabidopsis thaliana*
RcCCHC19	MRE	AACCTAA	*Petroselinum crispum*
RcCCHC19	MYB	CAACCA	*Arabidopsis thaliana*
RcCCHC19	MYC	CATTTG	*Arabidopsis thaliana*
RcCCHC19	TCA-element	CCATCTTTTT	*Nicotiana tabacum*
RcCCHC19	TC-rich repeats	ATTCTCTAAC	*Nicotiana tabacum*
RcCCHC19	TGA-element	AACGAC	*Brassica oleracea*
RcCCHC20	ABRE	CACGTG	*Arabidopsis thaliana*
RcCCHC20	ARE	AAACCA	*Zea mays*
RcCCHC20	as-1	TGACG	*Arabidopsis thaliana*
RcCCHC20	CGTCA-motif	CGTCA	*Hordeum vulgare*
RcCCHC20	DRE core	GCCGAC	*Arabidopsis thaliana*
RcCCHC20	GA-motif	ATAGATAA	*Arabidopsis thaliana*
RcCCHC20	G-box	CACGTG	*Arabidopsis thaliana*
RcCCHC20	MYB	CAACAG	*Arabidopsis thaliana*
RcCCHC20	MYC	CATGTG	*Arabidopsis thaliana*
RcCCHC20	P-box	CCTTTTG	*Oryza sativa*
RcCCHC20	TCA-element	CCATCTTTTT	*Nicotiana tabacum*
RcCCHC20	TGACG-motif	TGACG	*Hordeum vulgare*
RcCCHC21	ABRE	ACGTG	*Arabidopsis thaliana*
RcCCHC21	ARE	AAACCA	*Zea mays*
RcCCHC21	CAT-box	GCCACT	*Arabidopsis thaliana*
RcCCHC21	ERE	ATTTCATA	*Nicotiana glutinos*
RcCCHC21	G-box	CACGTC	*Zea mays*
RcCCHC21	GCN4_motif	TGAGTCA	*Oryza sativa*
RcCCHC21	LTR	CCGAAA	*Hordeum vulgare*
RcCCHC21	MRE	AACCTAA	*Petroselinum crispum*
RcCCHC21	MYB	CAACCA	*Arabidopsis thaliana*
RcCCHC21	MYC	CATTTG	*Arabidopsis thaliana*
RcCCHC21	STRE	AGGGG	*Arabidopsis thaliana*
RcCCHC21	TC-rich repeats	GTTTTCTTAC	*Nicotiana tabacum*
RcCCHC21	TGA-element	AACGAC	*Brassica oleracea*
RcCCHC21	WUN-motif	AAATTACT	*Nicotiana glutinosa*

RcCCHC22	A-box	CCGTCC	*Petroselinum crispum*
RcCCHC22	ARE	AAACCA	*Zea mays*
RcCCHC22	as-1	TGACG	*Arabidopsis thaliana*
RcCCHC22	Box 4	ATTAAT	*Petroselinum crispum*
RcCCHC22	CAT-box	GCCACT	*Arabidopsis thaliana*
RcCCHC22	CGTCA-motif	CGTCA	*Hordeum vulgare*
RcCCHC22	ERE	ATTTCATA	*Nicotiana glutinos*
RcCCHC22	G-box	CACGAC	*Zea mays*
RcCCHC22	GT1-motif	GGTTAA	*Arabidopsis thaliana*
RcCCHC22	LTR	CCGAAA	*Hordeum vulgare*
RcCCHC22	MYB	CAACCA	*Arabidopsis thaliana*
RcCCHC22	MYC	CATGTG	*Arabidopsis thaliana*
RcCCHC22	STRE	AGGGG	*Arabidopsis thaliana*
RcCCHC22	TCA-element	CCATCTTTTT	*Nicotiana tabacum*
RcCCHC22	TGACG-motif	TGACG	*Hordeum vulgare*
RcCCHC22	WUN-motif	AAATTACT	*Nicotiana glutinosa*
RcCCHC23	ARE	AAACCA	*Zea mays*
RcCCHC23	Box 4	ATTAAT	*Petroselinum crispum*
RcCCHC23	CAT-box	GCCACT	*Arabidopsis thaliana*
RcCCHC23	G-box	CACGAC	*Zea mays*
RcCCHC23	GT1-motif	GGTTAAT	*Avena sativa*
RcCCHC23	MBS	CAACTG	*Arabidopsis thaliana*
RcCCHC23	MYB	CAACAG	*Arabidopsis thaliana*
RcCCHC23	MYC	CATTTG	*Arabidopsis thaliana*
RcCCHC23	TCA-element	CCATCTTTTT	*Nicotiana tabacum*
RcCCHC23	W box	TTGACC	*Arabidopsis thaliana*
RcCCHC23	WUN-motif	AAATTTCTT	*Nicotiana glutinosa*
RcCCHC24	ABRE	GACACGTGGC	*Triticum aestivum*
RcCCHC24	ARE	AAACCA	*Zea mays*
RcCCHC24	as-1	TGACG	*Arabidopsis thaliana*
RcCCHC24	CGTCA-motif	CGTCA	*Hordeum vulgare*
RcCCHC24	GT1-motif	GGTTAA	*Arabidopsis thaliana*
RcCCHC24	I-box	atGATAAGGTC	*Helianthus annuus*
RcCCHC24	MBS	CAACTG	*Arabidopsis thaliana*
RcCCHC24	MYB	CAACAG	*Arabidopsis thaliana*
RcCCHC24	MYC	CATGTG	*Arabidopsis thaliana*
RcCCHC24	TGACG-motif	TGACG	*Hordeum vulgare*
RcCCHC25	A-box	CCGTCC	*Petroselinum crispum*
RcCCHC25	ABRE	ACGTG	*Arabidopsis thaliana*
RcCCHC25	ARE	AAACCA	*Zea mays*
RcCCHC25	as-1	TGACG	*Arabidopsis thaliana*
RcCCHC25	Box 4	ATTAAT	*Petroselinum crispum*
RcCCHC25	CAT-box	GCCACT	*Arabidopsis thaliana*
RcCCHC25	CGTCA-motif	CGTCA	*Hordeum vulgare*

RcCCHC25	GATA-motif	GATAGGA	*Arabidopsis thaliana*
RcCCHC25	G-Box	CACGTT	*Pisum sativum*
RcCCHC25	GT1-motif	GGTTAA	*Arabidopsis thaliana*
RcCCHC25	I-box	AGATAAGG	*Triticum aestivum*
RcCCHC25	LTR	CCGAAA	*Hordeum vulgare*
RcCCHC25	MBS	CAACTG	*Arabidopsis thaliana*
RcCCHC25	MRE	AACCTAA	*Petroselinum crispum*
RcCCHC25	MYB	CAACAG	*Arabidopsis thaliana*
RcCCHC25	MYC	CAATTG	*Arabidopsis thaliana*
RcCCHC25	P-box	CCTTTTG	*Oryza sativa*
RcCCHC25	STRE	AGGGG	*Arabidopsis thaliana*
RcCCHC25	TATC-box	TATCCCA	*Oryza sativa*
RcCCHC25	TCA-element	CCATCTTTTT	*Nicotiana tabacum*
RcCCHC25	TGACG-motif	TGACG	*Hordeum vulgare*
RcCCHC25	W box	TTGACC	*Arabidopsis thaliana*
RcCCHC25	WUN-motif	AAATTACT	*Nicotiana glutinosa*
RcCCHC26	ABRE	ACGTG	*Arabidopsis thaliana*
RcCCHC26	ARE	AAACCA	*Zea mays*
RcCCHC26	as-1	TGACG	*Arabidopsis thaliana*
RcCCHC26	Box 4	ATTAAT	*Petroselinum crispum*
RcCCHC26	CARE	CAACTCCC	*Oryza sativa*
RcCCHC26	CAT-box	GCCACT	*Arabidopsis thaliana*
RcCCHC26	CGTCA-motif	CGTCA	*Hordeum vulgare*
RcCCHC26	ERE	ATTTTAAA	*Nicotiana glutinos*
RcCCHC26	GATA-motif	GATAGGA	*Arabidopsis thaliana*
RcCCHC26	G-Box	CACGTT	*Pisum sativum*
RcCCHC26	MBS	CAACTG	*Arabidopsis thaliana*
RcCCHC26	MYB	CAACCA	*Arabidopsis thaliana*
RcCCHC26	MYC	CATTTG	*Arabidopsis thaliana*
RcCCHC26	TCA-element	TCAGAAGAGG	*Brassica oleracea*
RcCCHC26	TGACG-motif	TGACG	*Hordeum vulgare*
RcCCHC26	W box	TTGACC	*Arabidopsis thaliana*
RcCCHC27	ABRE	ACGTG	*Arabidopsis thaliana*
RcCCHC27	ARE	AAACCA	*Zea mays*
RcCCHC27	CARE	CAACTCAC	*Oryza sativa*
RcCCHC27	CAT-box	GCCACT	*Arabidopsis thaliana*
RcCCHC27	ERE	ATTTTAAA	*Nicotiana glutinos*
RcCCHC27	GATA-motif	AAGGATAAGG	*Solanum tuberosum*
RcCCHC27	G-box	TACGTG	*Arabidopsis thaliana*
RcCCHC27	MBS	CAACTG	*Arabidopsis thaliana*
RcCCHC27	MRE	AACCTAA	*Petroselinum crispum*
RcCCHC27	MYB	CAACCA	*Arabidopsis thaliana*
RcCCHC27	MYC	CATTTG	*Arabidopsis thaliana*
RcCCHC27	STRE	AGGGG	*Arabidopsis thaliana*

RcCCHC27	TCA-element	CCATCTTTTT	*Nicotiana tabacum*
RcCCHC27	W box	TTGACC	*Arabidopsis thaliana*
RcCCHC27	WUN-motif	AAATTTCTT	*Nicotiana glutinosa*
RcCCHC28	ABRE	ACGTG	*Arabidopsis thaliana*
RcCCHC28	ARE	AAACCA	*Zea mays*
RcCCHC28	as-1	TGACG	*Arabidopsis thaliana*
RcCCHC28	Box 4	ATTAAT	*Petroselinum crispum*
RcCCHC28	CAT-box	GCCACT	*Arabidopsis thaliana*
RcCCHC28	CGTCA-motif	CGTCA	*Hordeum vulgare*
RcCCHC28	G-box	TACGTG	*Arabidopsis thaliana*
RcCCHC28	I-box	cCATATCCAAT	*Flaveria trinervia*
RcCCHC28	MBS	CAACTG	*Arabidopsis thaliana*
RcCCHC28	MYB	CAACAG	*Arabidopsis thaliana*
RcCCHC28	MYC	CATTTG	*Arabidopsis thaliana*
RcCCHC28	STRE	AGGGG	*Arabidopsis thaliana*
RcCCHC28	TCA-element	TCAGAAGAGG	*Brassica oleracea*
RcCCHC28	TC-rich repeats	GTTTTCTTAC	*Nicotiana tabacum*
RcCCHC28	TGACG-motif	TGACG	*Hordeum vulgare*
RcCCHC28	W box	TTGACC	*Arabidopsis thaliana*
RcCCHC28	WUN-motif	AAATTTCTT	*Nicotiana glutinosa*
RcCCHC29	ABRE	CACGTG	*Arabidopsis thaliana*
RcCCHC29	Box 4	ATTAAT	*Petroselinum crispum*
RcCCHC29	circadian	CAAAGATATC	*Lycopersicon esculentum*
RcCCHC29	ERE	ATTTCATA	*Nicotiana glutinos*
RcCCHC29	G-Box	CACGTG	*Pisum sativum*
RcCCHC29	GT1-motif	GGTTAA	*Arabidopsis thaliana*
RcCCHC29	I-box	atGATAAGGTC	*Helianthus annuus*
RcCCHC29	MBS	CAACTG	*Arabidopsis thaliana*
RcCCHC29	MYB	CAACCA	*Arabidopsis thaliana*
RcCCHC29	MYC	CATTTG	*Arabidopsis thaliana*
RcCCHC29	TC-rich repeats	ATTCTCTAAC	*Nicotiana tabacum*
RcCCHC29	W box	TTGACC	*Arabidopsis thaliana*
RcCCHC30	A-box	CCGTCC	*Petroselinum crispum*
RcCCHC30	ABRE	ACGTG	*Arabidopsis thaliana*
RcCCHC30	as-1	TGACG	*Arabidopsis thaliana*
RcCCHC30	Box 4	ATTAAT	*Petroselinum crispum*
RcCCHC30	CGTCA-motif	CGTCA	*Hordeum vulgare*
RcCCHC30	circadian	CAAAGATATC	*Lycopersicon esculentum*
RcCCHC30	GA-motif	ATAGATAA	*Arabidopsis thaliana*
RcCCHC30	GATA-motif	AAGATAAGATT	*Arabidopsis thaliana*
RcCCHC30	G-box	CACGTC	*Zea mays*
RcCCHC30	GT1-motif	GGTTAA	*Arabidopsis thaliana*
RcCCHC30	MYB	TAACCA	*Arabidopsis thaliana*
RcCCHC30	MYC	CATTTG	*Arabidopsis thaliana*

RcCCHC30	STRE	AGGGG	*Arabidopsis thaliana*
RcCCHC30	TATC-box	TATCCCA	*Oryza sativa*
RcCCHC30	TGA-element	AACGAC	*Brassica oleracea*
RcCCHC30	W box	TTGACC	*Arabidopsis thaliana*
RcCCHC30	WUN-motif	AAATTACT	*Nicotiana glutinosa*
RcCCHC31	ABRE	ACGTG	*Arabidopsis thaliana*
RcCCHC31	ARE	AAACCA	*Zea mays*
RcCCHC31	Box 4	ATTAAT	*Petroselinum crispum*
RcCCHC31	CAT-box	GCCACT	*Arabidopsis thaliana*
RcCCHC31	G-box	TACGTG	*Arabidopsis thaliana*
RcCCHC31	I-box	GTATAAGGCC	*Larix laricina*
RcCCHC31	MBS	CAACTG	*Arabidopsis thaliana*
RcCCHC31	MYB	TAACCA	*Arabidopsis thaliana*
RcCCHC31	MYC	CATTTG	*Arabidopsis thaliana*
RcCCHC31	STRE	AGGGG	*Arabidopsis thaliana*
RcCCHC31	TC-rich repeats	GTTTTCTTAC	*Nicotiana tabacum*
RcCCHC31	W box	TTGACC	*Arabidopsis thaliana*
RcCCHC32	ABRE	ACGTG	*Arabidopsis thaliana*
RcCCHC32	ARE	AAACCA	*Zea mays*
RcCCHC32	as-1	TGACG	*Arabidopsis thaliana*
RcCCHC32	Box 4	ATTAAT	*Petroselinum crispum*
RcCCHC32	CARE	CAACTCCC	*Oryza sativa*
RcCCHC32	CAT-box	GCCACT	*Arabidopsis thaliana*
RcCCHC32	CGTCA-motif	CGTCA	*Hordeum vulgare*
RcCCHC32	circadian	CAAAGATATC	*Lycopersicon esculentum*
RcCCHC32	GA-motif	ATAGATAA	*Arabidopsis thaliana*
RcCCHC32	GATA-motif	AAGGATAAGG	*Solanum tuberosum*
RcCCHC32	G-Box	CACGTG	*Pisum sativum*
RcCCHC32	GT1-motif	GGTTAA	*Arabidopsis thaliana*
RcCCHC32	LTR	CCGAAA	*Hordeum vulgare*
RcCCHC32	MBS	CAACTG	*Arabidopsis thaliana*
RcCCHC32	MYB	CAACCA	*Arabidopsis thaliana*
RcCCHC32	MYC	CATTTG	*Arabidopsis thaliana*
RcCCHC32	P-box	CCTTTTG	*Oryza sativa*
RcCCHC32	STRE	AGGGG	*Arabidopsis thaliana*
RcCCHC32	TGACG-motif	TGACG	*Hordeum vulgare*
RcCCHC32	TGA-element	AACGAC	*Brassica oleracea*
RcCCHC32	W box	TTGACC	*Arabidopsis thaliana*
RcCCHC33	A-box	CCGTCC	*Petroselinum crispum*
RcCCHC33	ABRE	ACGTG	*Arabidopsis thaliana*
RcCCHC33	ARE	AAACCA	*Zea mays*
RcCCHC33	as-1	TGACG	*Arabidopsis thaliana*
RcCCHC33	Box 4	ATTAAT	*Petroselinum crispum*
RcCCHC33	CARE	CAACTCCC	*Oryza sativa*

RcCCHC33	CAT-box	GCCACT	*Arabidopsis thaliana*
RcCCHC33	CGTCA-motif	CGTCA	*Hordeum vulgare*
RcCCHC33	DRE core	GCCGAC	*Arabidopsis thaliana*
RcCCHC33	GATA-motif	GATAGGG	*Pisum sativum*
RcCCHC33	G-box	TACGTG	*Arabidopsis thaliana*
RcCCHC33	GT1-motif	GGTTAA	*Arabidopsis thaliana*
RcCCHC33	I-box	atGATAAGGTC	*Helianthus annuus*
RcCCHC33	MBS	CAACTG	*Arabidopsis thaliana*
RcCCHC33	MYB	CAACCA	*Arabidopsis thaliana*
RcCCHC33	MYC	CATGTG	*Arabidopsis thaliana*
RcCCHC33	P-box	CCTTTTG	*Oryza sativa*
RcCCHC33	STRE	AGGGG	*Arabidopsis thaliana*
RcCCHC33	TGACG-motif	TGACG	*Hordeum vulgare*
RcCCHC34	ABRE	CACGTG	*Arabidopsis thaliana*
RcCCHC34	ARE	AAACCA	*Zea mays*
RcCCHC34	as-1	TGACG	*Arabidopsis thaliana*
RcCCHC34	Box 4	ATTAAT	*Petroselinum crispum*
RcCCHC34	CGTCA-motif	CGTCA	*Hordeum vulgare*
RcCCHC34	ERE	ATTTTAAA	*Nicotiana glutinos*
RcCCHC34	G-box	CACGTG	*Arabidopsis thaliana*
RcCCHC34	I-box	cCATATCCAAT	*Flaveria trinervia*
RcCCHC34	LTR	CCGAAA	*Hordeum vulgare*
RcCCHC34	MBS	CAACTG	*Arabidopsis thaliana*
RcCCHC34	MYB	CAACCA	*Arabidopsis thaliana*
RcCCHC34	MYC	CATTTG	*Arabidopsis thaliana*
RcCCHC34	STRE	AGGGG	*Arabidopsis thaliana*
RcCCHC34	TC-rich repeats	GTTTTCTTAC	*Nicotiana tabacum*
RcCCHC34	TGACG-motif	TGACG	*Hordeum vulgare*
RcCCHC34	W box	TTGACC	*Arabidopsis thaliana*
RcCCHC35	ABRE	ACGTG	*Arabidopsis thaliana*
RcCCHC35	ARE	AAACCA	*Zea mays*
RcCCHC35	Box 4	ATTAAT	*Petroselinum crispum*
RcCCHC35	GATA-motif	AAGGATAAGG	*Solanum tuberosum*
RcCCHC35	G-Box	CACGTT	*Pisum sativum*
RcCCHC35	GCN4_motif	TGAGTCA	*Oryza sativa*
RcCCHC35	LTR	CCGAAA	*Hordeum vulgare*
RcCCHC35	MBS	CAACTG	*Arabidopsis thaliana*
RcCCHC35	MYB	TAACCA	*Arabidopsis thaliana*
RcCCHC35	MYC	CATGTG	*Arabidopsis thaliana*
RcCCHC35	W box	TTGACC	*Arabidopsis thaliana*
RcCCHC36	ARE	AAACCA	*Zea mays*
RcCCHC36	as-1	TGACG	*Arabidopsis thaliana*
RcCCHC36	Box 4	ATTAAT	*Petroselinum crispum*
RcCCHC36	CAT-box	GCCACT	*Arabidopsis thaliana*

RcCCHC36	CGTCA-motif	CGTCA	*Hordeum vulgare*
RcCCHC36	circadian	CAAAGATATC	*Lycopersicon esculentum*
RcCCHC36	GATA-motif	AAGATAAGATT	*Arabidopsis thaliana*
RcCCHC36	G-box	CACGAC	*Zea mays*
RcCCHC36	I-box	AAGATAAGGCT	*Gossypium hirsutum*
RcCCHC36	MBS	CAACTG	*Arabidopsis thaliana*
RcCCHC36	MRE	AACCTAA	*Petroselinum crispum*
RcCCHC36	MYB	CAACCA	*Arabidopsis thaliana*
RcCCHC36	MYC	CATTTG	*Arabidopsis thaliana*
RcCCHC36	TGACG-motif	TGACG	*Hordeum vulgare*
RcCCHC36	W box	TTGACC	*Arabidopsis thaliana*
RcCCHC37	ABRE	ACGTG	*Arabidopsis thaliana*
RcCCHC37	ARE	AAACCA	*Zea mays*
RcCCHC37	Box 4	ATTAAT	*Petroselinum crispum*
RcCCHC37	ERE	ATTTCATA	*Nicotiana glutinos*
RcCCHC37	G-Box	CACGTT	*Pisum sativum*
RcCCHC37	GT1-motif	GGTTAAT	*Avena sativa*
RcCCHC37	I-box	atGATAAGGTC	*Helianthus annuus*
RcCCHC37	LTR	CCGAAA	*Hordeum vulgare*
RcCCHC37	MYB	CAACCA	*Arabidopsis thaliana*
RcCCHC37	MYC	CATGTG	*Arabidopsis thaliana*
RcCCHC37	STRE	AGGGG	*Arabidopsis thaliana*
RcCCHC37	TATC-box	TATCCCA	*Oryza sativa*
RcCCHC37	TC-rich repeats	GTTTTCTTAC	*Nicotiana tabacum*
RcCCHC37	W box	TTGACC	*Arabidopsis thaliana*
RcCCHC37	WUN-motif	TTATTACAT	*Nicotiana glutinosa*
RcCCHC38	ABRE	ACGTG	*Arabidopsis thaliana*
RcCCHC38	ARE	AAACCA	*Zea mays*
RcCCHC38	as-1	TGACG	*Arabidopsis thaliana*
RcCCHC38	Box 4	ATTAAT	*Petroselinum crispum*
RcCCHC38	CGTCA-motif	CGTCA	*Hordeum vulgare*
RcCCHC38	circadian	CAAAGATATC	*Lycopersicon esculentum*
RcCCHC38	ERE	ATTTCATA	*Nicotiana glutinos*
RcCCHC38	GATA-motif	GATAGGG	*Pisum sativum*
RcCCHC38	G-box	TACGTG	*Arabidopsis thaliana*
RcCCHC38	GT1-motif	GGTTAA	*Arabidopsis thaliana*
RcCCHC38	LTR	CCGAAA	*Hordeum vulgare*
RcCCHC38	MBS	CAACTG	*Arabidopsis thaliana*
RcCCHC38	MRE	AACCTAA	*Petroselinum crispum*
RcCCHC38	MYB	CAACAG	*Arabidopsis thaliana*
RcCCHC38	MYC	CATTTG	*Arabidopsis thaliana*
RcCCHC38	STRE	AGGGG	*Arabidopsis thaliana*
RcCCHC38	TCA-element	TCAGAAGAGG	*Brassica oleracea*
RcCCHC38	TGACG-motif	TGACG	*Hordeum vulgare*

RcCCHC38	W box	TTGACC	*Arabidopsis thaliana*
RcCCHC39	ABRE	ACGTG	*Arabidopsis thaliana*
RcCCHC39	ARE	AAACCA	*Zea mays*
RcCCHC39	Box 4	ATTAAT	*Petroselinum crispum*
RcCCHC39	CAT-box	GCCACT	*Arabidopsis thaliana*
RcCCHC39	DRE core	GCCGAC	*Arabidopsis thaliana*
RcCCHC39	G-box	CAGACGTGGCA	*Nicotiana plumbaginifolia*
RcCCHC39	LTR	CCGAAA	*Hordeum vulgare*
RcCCHC39	MYB	CAACCA	*Arabidopsis thaliana*
RcCCHC39	MYC	CATGTG	*Arabidopsis thaliana*
RcCCHC39	STRE	AGGGG	*Arabidopsis thaliana*
RcCCHC39	W box	TTGACC	*Arabidopsis thaliana*
RcCCHC39	WUN-motif	AAATTTCCT	*Brassica oleracea*
RcCCHC40	A-box	CCGTCC	*Petroselinum crispum*
RcCCHC40	ABRE	ACGTG	*Arabidopsis thaliana*
RcCCHC40	as-1	TGACG	*Arabidopsis thaliana*
RcCCHC40	CAT-box	GCCACT	*Arabidopsis thaliana*
RcCCHC40	CGTCA-motif	CGTCA	*Hordeum vulgare*
RcCCHC40	circadian	CAAAGATATC	*Lycopersicon esculentum*
RcCCHC40	DRE core	GCCGAC	*Arabidopsis thaliana*
RcCCHC40	G-box	CAGACGTGGCA	*Nicotiana plumbaginifolia*
RcCCHC40	GT1-motif	GGTTAAT	*Avena sativa*
RcCCHC40	MYB	CAACCA	*Arabidopsis thaliana*
RcCCHC40	MYC	CATTTG	*Arabidopsis thaliana*
RcCCHC40	P-box	CCTTTTG	*Oryza sativa*
RcCCHC40	STRE	AGGGG	*Arabidopsis thaliana*
RcCCHC40	TGACG-motif	TGACG	*Hordeum vulgare*
RcCCHC40	TGA-element	AACGAC	*Brassica oleracea*
RcCCHC40	W box	TTGACC	*Arabidopsis thaliana*
RcCCHC40	WUN-motif	AAATTTCCT	*Brassica oleracea*
RcCCHC41	A-box	CCGTCC	*Petroselinum crispum*
RcCCHC41	ARE	AAACCA	*Zea mays*
RcCCHC41	Box 4	ATTAAT	*Petroselinum crispum*
RcCCHC41	CARE	CAACTCAC	*Oryza sativa*
RcCCHC41	circadian	CAAAGATATC	*Lycopersicon esculentum*
RcCCHC41	GT1-motif	GGTTAA	*Arabidopsis thaliana*
RcCCHC41	I-box	cCATATCCAAT	*Flaveria trinervia*
RcCCHC41	MYB	CAACCA	*Arabidopsis thaliana*
RcCCHC41	MYC	CATTTG	*Arabidopsis thaliana*
RcCCHC41	P-box	CCTTTTG	*Oryza sativa*
RcCCHC41	STRE	AGGGG	*Arabidopsis thaliana*
RcCCHC41	TATC-box	TATCCCA	*Oryza sativa*
RcCCHC41	TCA-element	TCAGAAGAGG	*Brassica oleracea*
RcCCHC41	W box	TTGACC	*Arabidopsis thaliana*

RcCCHC41	WUN-motif	AAATTTCCT	*Brassica oleracea*

Table S6. Reference genome source.

Species	Genome Source
Rosa chinensis 'Old Blush'	https://lipm-browsers.toulouse.inra.fr/pub/RchiOBHm-V2/
Ginkgo biloba	https://ginkgo.zju.edu.cn/genome/
Ostreococcus lucimarinus	https://www.ncbi.nlm.nih.gov/datasets/genome/GCF_000092065.1/
Arabidopsis thaliana	https://plants.ensembl.org/Arabidopsis_thaliana/Info/Index
Oryza sativa subsp. *japonica*	https://plants.ensembl.org/Oryza_sativa/Info/Index
Triticum aestivum	https://plants.ensembl.org/Triticum_aestivum/Info/Index
Vitis vinifera	https://plants.ensembl.org/Vitis_vinifera/Info/Index
Fragaria ananassa	https://www.rosaceae.org/species/fragaria/all
Prunus persica	https://www.rosaceae.org/Analysis/13087664
Rosa rugosa	https://ngdc.cncb.ac.cn/gwh/Genome/496/show
Solanum lycopersicum	https://ngdc.cncb.ac.cn/gwh/Assembly/18677/show

Table S7. Identified duplication gene pairs in the *CCHC* gene family of *Rosa chinensis*.

	Gene1	Gene2
WGD[1]	*RcCCHC7*	*RcCCHC20*
	RcCCHC14	*RcCCHC30*
PD[2]	*RcCCHC39*	*RcCCHC40*
TRD[3]	*RcCCHC4*	*RcCCHC14*
	RcCCHC17	*RcCCHC5*
	RcCCHC31	*RcCCHC10*
	RcCCHC33	*RcCCHC14*
DSD[4]	*RcCCHC1*	*RcCCHC12*
	RcCCHC35	*RcCCHC1*
	RcCCHC1	*RcCCHC36*
	RcCCHC2	*RcCCHC41*
	RcCCHC9	*RcCCHC32*
	RcCCHC41	*RcCCHC9*
	RcCCHC12	*RcCCHC36*
	RcCCHC22	*RcCCHC15*
	RcCCHC27	*RcCCHC15*
	RcCCHC28	*RcCCHC15*
	RcCCHC27	*RcCCHC22*
	RcCCHC22	*RcCCHC28*
	RcCCHC24	*RcCCHC29*
	RcCCHC24	*RcCCHC37*
	RcCCHC25	*RcCCHC34*
	RcCCHC27	*RcCCHC28*
	RcCCHC29	*RcCCHC37*
	RcCCHC35	*RcCCHC36*

[1, 2, 3, 4] In this table, WGD, PD, TRD, and DSD represent whole gene duplication, proximal duplication, retrotransposed duplication, and disperse duplication, respectively.

Table S8. List of primers used in this study.

Name of primer	Primer sequence (5'-3')
qRcCCHC25-F	TGAACCTGGTCACTTTGCT
qRcCCHC25-R	TCGTAACTGGGACTTGTGC
qRcCCHC28-F	CCTACTGTGGCACTTATCTTT
qRcCCHC28-R	CCATCTCATCACTGCTCCC
qRcCCHC9-F	CAACAAATGTAAACGACCAGG
qRcCCHC9-R	TTGCAGTTCCAACAAGTAGCG
qRcCCHC7-F	CCGTGAGTATCTTGGTAGAGGAC
qRcCCHC7-R	TGACAATTTCTTTCAATGTGGC
qRcCCHC20-F	GCTGCTTTAATTGTGGCATTG
qRcCCHC20-R	CGCTCACGCCTTAGTTCTTTT
qRcCCHC25-F	CGCACAAGTCCCAGTTACGAT
qRcCCHC25-R	CAGTCCCCATTGGCATAAGGT
qRcCCHC14-F	ACCAAGGGTTTCGGCTTCATC
qRcCCHC14-R	CCGTTCGGACCAGTGACATCG
qRcUBI2-F	GCCCTGGTGCGTTCCCAACTG
qRcUBI2-R	CCTGCGTGTCTGTCCGCATTG
RcCCHC25V-F	GCACAAGTCCCAGTTACGATG
RcCCHC25V-R	CGAACAGAACGCATTGTCATAAC
RcCCHC25-TRV2-F	gtgagtaaggttaccgaattcAGGCGCAGAAGTCCTTCCC
RcCCHC25-TRV2-R	cgtgagctcggtaccggatccCGAACAGAACGCATTGTCATAAC

Table S9. List of proteins and gene sequences in this study.

Gene ID	Sequence (5' to 3')
	**mRNA sequence**
RcCCHC25	CTTCTCTATCCGATCGATCATCCAAGGACGATGACTCGCGTTTACGTTGGGAACTTGGATCCTCGA<br>GTGAGTGATAGGGATCTTGAAGATGAGTTTCGCATGTTCGGCGTTCTTCGCAGTGTGTGGGTTGCT<br>CGGAGGCCACCGGGATATGCCTTTGTTGAATTTGATGACCACAGGGATGCTCTTGATGCCATTCA<br>GAGATTGGATGGAAAAAATGGTTGGCGTGTGGAACTGTCCCATAACTCTAAGGGTGGTGGTGGT<br>GGTGGTGGAGGCCGTGGCGGGCGTGGTGGAGGTGACGACTTGAAGTGTTATGAATGTGGTGAAC<br>CTGGTCACTTTGCTCGAGAGTGTCGTATGCGTGTTGGTTCACGAGGATTGGGTAGCGGAAGGCGC<br>AGAAGTCCTTCCCCTCGACGTGGCCGCACAAGTCCCAGTTACGATGGTTATGGGCATGGGCGCAG<br>GACTTATAGTCCACGTAGGAGGAGATCTCCTCCTCTACGACGTCGCACCCCATCACCTCTGCCTCG<br>ACGTGGACGCAGCTACAGCAGGTCTCCCCCATACCGCCATGCTCGACGTGTTTCACCTTATGCCA<br>ATGGGGACTGAGCTGGAACTTGATTTGAGCAAATGCTGGATGGATGTTGAAAGGCTGATATCTTC<br>CTGCTATAAACTAGTTCTAATAGGATTGGTTGAACTGACAAGAACCATGTTAGTTATGTTGATGA<br>TGTTATGACAATGCGTTCTGTTCGAAG
	**protein sequence**
	MTRVYVGNLDPRVSDRDLEDEFRMFGVLRSVWVARRPPGYAFVEFDDHRDALDAIQRLDGKNGWR<br>VELSHNSKGGGGGGGGRGGRGGGDDLKCYECGEPGHFARECRMRVGSRGLGSGRRRSPSPRRGRTS<br>PSYDGYGHGRRTYSPRRRRSPPLRRRTPSPLPRRGRSYSRSPPYRHARR
